# Evaluation of proprioception and postural control at a minimum 1 year follow-up after ankle capsuloligamentous lateralplasty with Brostrom technique: A cohort study

**DOI:** 10.1097/MD.0000000000019862

**Published:** 2020-04-24

**Authors:** Massimiliano Mosca, Silvio Caravelli, Simone Massimi, Mario Fuiano, Giuseppe Catanese, Giuseppe Barone, Laura Bragonzoni, Maria Grazia Benedetti

**Affiliations:** a II Clinic of Orthopaedics and Traumatology; b Dipartimento delle Scienze per la Qualità della Vita; c Dipartimento delle Scienze Biomediche e Neuromotorie; d Physical Medicine and Rehabilitation Unit, IRCCS Istituto Ortopedico Rizzoli, Bologna, Italy.

**Keywords:** ankle sprain, Brostrom, Chronic Ankle Instability, postural balance, proprioception

## Abstract

Recovery of postural control and proprioception in patients affected by chronic ankle instability (CAI) and operated on capsulo-ligaments reconstructive surgery lacks of objective assessment. The aim of this study was to evaluate long-term post-surgical postural and proprioceptive control through the DPPS device in a cohort of patients operated on ligaments reconstruction through the modified Brostrom procedure at a minimum follow up of 12 months.

Eleven patients with post-traumatic lateral CAI, operated of external capsulo-ligamentous complex repair according to Brostrom technique at a minimum follow-up of 1 year were enrolled. Physical examination and American Orthopaedics Foot and Ankle Society (AOFAS) ankle-hindfoot score. Proprioceptive and postural stability was assessed by DPPS - Delos Postural Proprioceptive System, linked to a computer with a specific software and including a flat table, an electronic unstable proprioceptive board, a Delos Vertical Controller, a monitor and a horizontal bar fitted with an infra-red sensor for hand support.

Patients were 5 males and 6 females, mean age of 38.4 ± 12 years. Mean BMI of the patients was 26.8 ± 4.4. Mean follow up was 13.4 ± 2.1. The mean value of (AOFAS) clinical score was 90.3/100. Mean Static Stability Index (SSI) with open eyes was 87.7% (±7.6) in the operated leg and 90.4% (±6.1) in the contra-lateral. SSI with closed eyes was 64.5% (±11.2) in the operated leg and 61.6% (±16.8) in the contra-lateral. Mean Dynamic Stability Index (DSI) without restrictions was 56.2% (±14.6) in the operated leg and 56.8% (±10.6) in the contra-lateral. DSI with restricted upper limbs, had a mean value of 56.3% (±11.4) in the operated leg and 58.1% (±11.9) in the contra-lateral.

Re-tensioning capsular-ligamentous surgery of the external compartment for CAI allow to recovery proprioceptive and postural control on the operated side, comparable with data from the contralateral limb and from the healthy population of the same age and sex.

## 1 Introduction

Ligamentous injuries of the ankle lateral complex are among the most common lesions experienced by athletes.^[1]^ The main predisposing factor to ankle trauma is a clinical history of sprain.^[1]^ People with recurrent ankle sprains are generally classified either as caused by functional instability or by chronic mechanical instability, generally referred as chronic lateral instability of the ankle (CAI).^[2–5]^ Previous study demonstrated that mechanical instability as a cause of CAI is due to pathologic laxity after ankle ligament injury and supposed to be responsible for balance deficits, proprioception impairment, and in particular abnormal joint position sense deficit, delayed peroneal muscle reaction times, strength deficits.^[6–9]^ Such poor postural control was associated to increased risk of recurrent ankle sprain. The modified Broström procedure is used for treating patients with mechanical ankle instability.^[10–11]^ However, studies on recovery of proprioception and postural stability after surgical ligamentous reconstruction are very limited^[6,12,13]^ and, mainly focused on the assessment of sense of position and postural instability.

Recently, a new device, the Delos Postural Proprioceptive System (DPPS; Delos, Turin, Italy) has been introduced by Riva et al^[2]^ to assess and train proprioception in professional basketball players with recurrent ankle sprains. In particular, the device allows to test the single stance stability, which is based on proprioceptive control (minimizing the visual and vestibular contribution) to guarantee the safety of basic movements such as walking, running, and jumping.^[14]^

The aim of this study was to evaluate long term post-surgical postural and proprioceptive control through the DPPS device in a cohort of patients operated on ligaments reconstruction through the modified Brostrom procedure at a minimum follow up of 12 months. The research hypothesis is that surgical repair of CAI could restore optimal proprioceptive and postural control both with respect to contralateral limb and to standard report for healthy subjects.

## 2 Methods

### 2.1 Study design, ethics, setting, sample size

The study was designed as a cohort descriptive study in which patients operated on modified Brostrom procedure at a minimum follow up of 1 year were recalled to be clinically evaluated in an outpatient setting where they were clinically evaluated and tested with the Delos Postural Proprioceptive System.

The study was approved on 12/20/2017 by the Central Emilia Wide Area Ethical Committee of the Emilia-Romagna Region (CE-AVEC) (protocol number 0012824). Data were collected and reviewed by 2 different and “blinded” surgeons, in order to avoid bias.

Clinical sheets of all the patients with post-traumatic lateral chronic instability of the ankle, who underwent surgical repair of the external capsular-ligamentous compartment by the same surgeon (M.M.), according to the modified Brostrom technique, without other ankle surgical procedures, and at a minimum follow up of 1 year, were retrieved from the hospital archives. Patients with chronic inflammatory joint diseases, pre-existing anomalies of gait kinematics and neurological diseases, mid-high-grade ankle or knee osteoarthritis, severe postural instability, and cognitive impairment were excluded.

### 2.2 Participants

**Figure 1:**
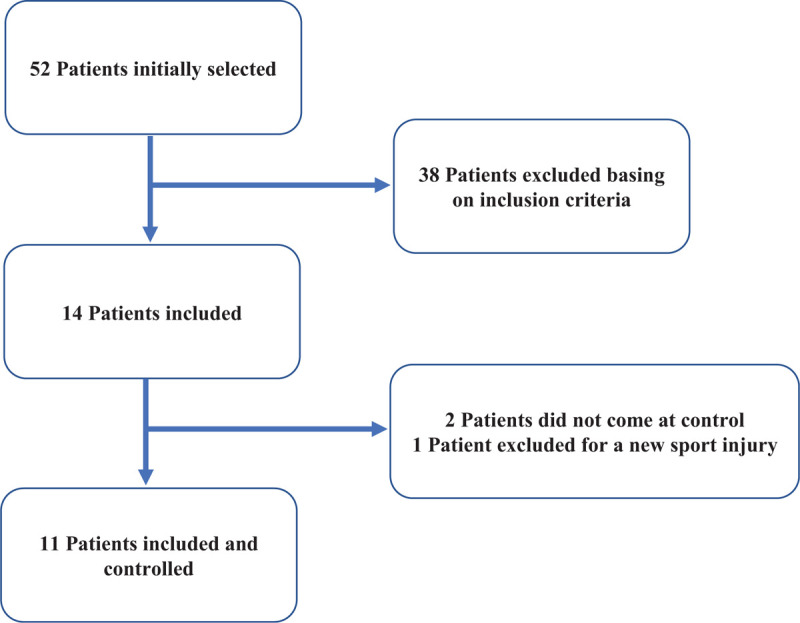
Patients flowchart.

Fifty two patients were identified. Of these only 14 were considered eligible for the study and contacted. Two patients were not able to come at the control because living far from the study site, and 1 patient was excluded for recent sport injury. Eleven patients were eventually included in the study and signed the informed consent (Fig. 1).

### 2.3 Intervention

#### 2.3.1 Surgical technique

Under spinal anesthesia, the patient was placed in lateral position, with feet at about 2 cm from the end of the operating table and a thigh tourniquet was applied and inflated for lower limb exsanguination, following the AORN (Association of periOperative Registered Nurses) guidelines (tourniquet inflated intraoperatively to a pressure higher than the limb occlusion pressure).

A lateral 4 to 4.5 cm vertical skin incision was performed anteriorly to the fibular malleolus, curving posteriorly and downwards following peroneal distal edge. After carefully avoiding to injury the superficial peroneal nerve, subcutis, and superficial retinaculum were dissected from the underlying capsule-ligamentous structures. Capsule and ATFL complex were detached from the antero-inferior edge of the malleolus and any loose body or bone fragment is removed. Within the joint capsule, the anterior talofibular ligament was identified as a thickening of the capsule fibular border, and was then roughened with a bone rongeur to improve soft tissue adherence. Three holes, oriented obliquely from proximal to distal and from lateral to medial, were created in fibular edge drilling with a 1.8 K-wire. Paying attention to maintain the foot pronated and abducted, trans-osseous suture between distal edge of the fibula and capsule-ligamentous complex was performed with 3 absorbable no. 2 wires. Additionally, the repair was reinforced by suturing a fibular periosteum flap over the abovementioned capsule-ligamentous tissue using interrupted absorbable no. 2.0 suture.

### 2.4 Outcomes of the study

Main outcome of the study was represented by the instrumental assessment of proprioception and postural stability through the Delos Postural Proprioceptive System.

Clinical ankle stability and functional status were evaluated as secondary end-point. All patients included in the study underwent physical examination through anterior drawer test and inversion tilt tests in order to evaluate the mechanical stability of the operated tibio-tarsal joint. In addition, clinical AOFAS (American Orthopaedic Foot and Ankle Society) ankle-hindfoot score was evaluated.

### 2.5 Instrumental evaluation

**Figure 2:**
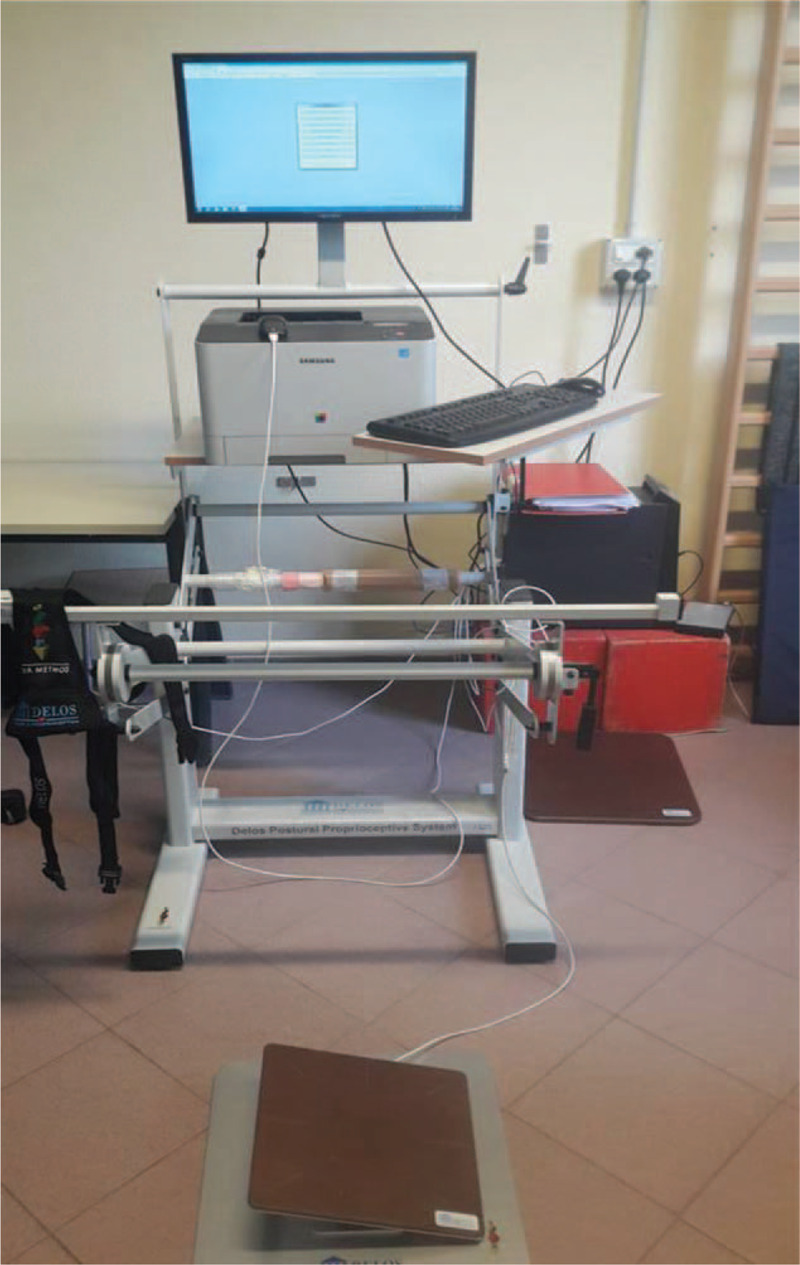
The DELOS Postural Proprioceptive System (DELOSs.r.l., Turin, Italy) was used for this study. The horizontal bar, the Delos Assistant Desk (DAD), fitted with an infra-red sensor, is set in front of the patient for hand support. The DAD measures the times and the duration of leaning the hands of the patient, expresses in terms of percentage providing information about the so called “precautionary strategy” that is the way the patients prevent falls, and regain the vertical posture.

Proprioceptive stability tests were performed by DPPS - Delos Postural Proprioceptive System (Delos S.R.L. – Turin, ITALY) at our Institute. The station is managed by a computer with a specific software (DPPS 6.0, latest version) and includes a flat table, an electronic Rocking Board (unstable proprioceptive board), a Delos Vertical Controller (postural electronic reader, DVC) and a monitor. A horizontal bar, the Delos Assistant Desk (DAD), fitted with an infra-red sensor, is set in front of the patient for hand support. The DAD measures how many times and how long a patient leans their hands for support. The measurement is expressed in terms of percentage, providing information on the so-called “precautionary strategy”, which is the way patients prevent falls, and regain vertical posture (Fig. 2). In the event of fall risk, the infrared bar set in front of the subject provides a potential point of support in order for the subject to grab and quickly recover vertical control; the bar would record every time it was used as support. The DVC, applied to the patient sternum, measures the inclination of the trunk in the frontal (x) and sagittal (y) plane by a bi-dimensional accelerometric unit. The unstable proprioceptive board has only 1 degree of freedom on the frontal plane and can detect the inclination versus the ground,^[11]^ which is visually represented on the monitor.

The tests usually performed are the static proprioceptive Riva test and the visual-proprioceptive dynamic Riva test.

**Figure 3:**
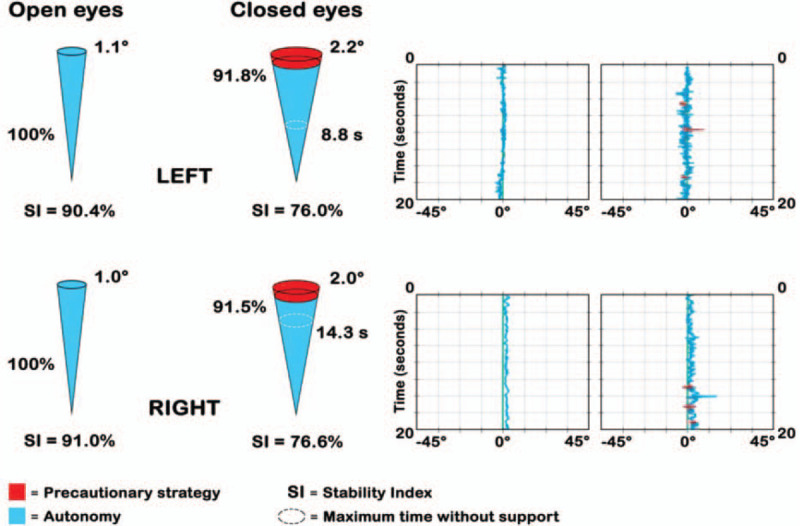
Graphic evaluation of the Static Stability Index (SSI). SSI is represented with cones (at the left), measuring its amplitude and using a color code to mean (1) BLU autonomy, i.e., the component without support and (2) RED precaution strategy. The time without support is also registered. RED and BLU are also expressed in % of total time (if strategy is < 10% → it is not significant). Clusters (at the right) represent oscillations of the body vs the x and y axes. In this test the subject does not receive postural feedback and every trial lasts 20 seconds.

In the *static proprioceptive Riva test*, the patient is asked to stay in single leg stance, alternating their right and left legs for a total of 4 tests; 2 with open eyes (OE) and 2 with closed eyes (CE). Each trial lasts for 20 seconds followed by a pause of 15 seconds. The Static Stability Index (SSI) is obtained with this test, expressed as a percentage and represents the functional level of the postural control with open eyes. The difference between this value and the SSI measured with closed eyes, gives an idea on the visual dependence, indicating the difference between global postural control of the patient (visual control-assisted proprioception) and the effective proprioception control (with closed eyes). The graphic evaluation of the SSI is made with cones of different amplitude and are color-coded (Fig. 3).

**Figure 4:**
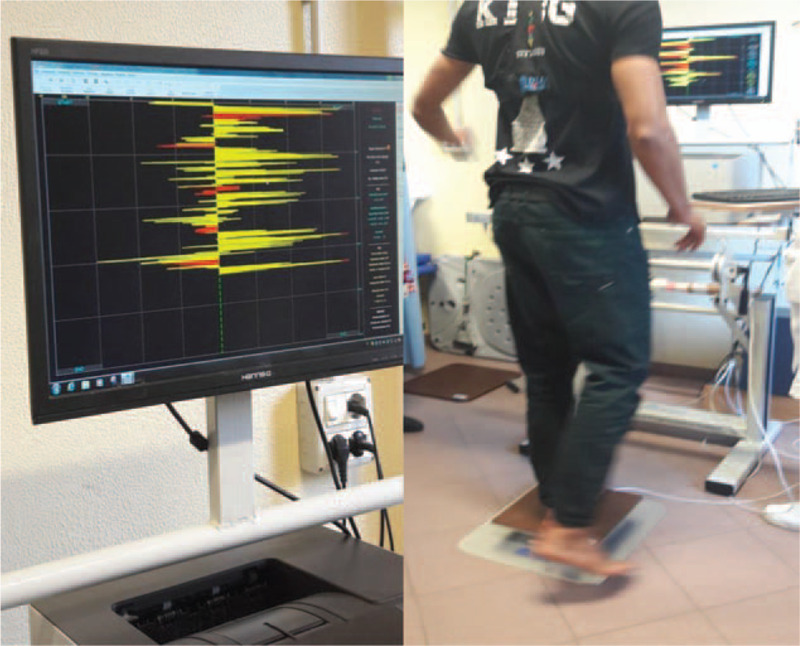
“Rocking Error” graphic representation during the visual-proprioceptive dynamic Riva test.

The *visual-proprioceptive dynamic Riva test* takes advantage of Delos Rocking Board. During the test, the patient is required to stay in balance on the Rocking board, in single leg stance in the middle of the board, trying to keep it as less inclined as possible on the horizontal plane. On the monitor, the subject is able to observe the Rocking Error (RE) in real time, i.e. the oscillation of the board represented as a yellow line separated from a central dotted line (Fig. 4). This visual feedback can help subjects to restore balance. The test includes 8 trials: 4 without restrictions and 4 with restrictions, alternating support on the right and left leg. The restriction in our case was placing string between the thumbs of subjects, allowing the arms to get close to the torso, minimizing the so-called secondary compensation (the attempt to balance the body moving the upper part) and unblocking the lower part. This is not only more efficient in postural control, but it also challenges the proprioceptive centers of the ankle and the lower leg in general.

**Figure 5:**
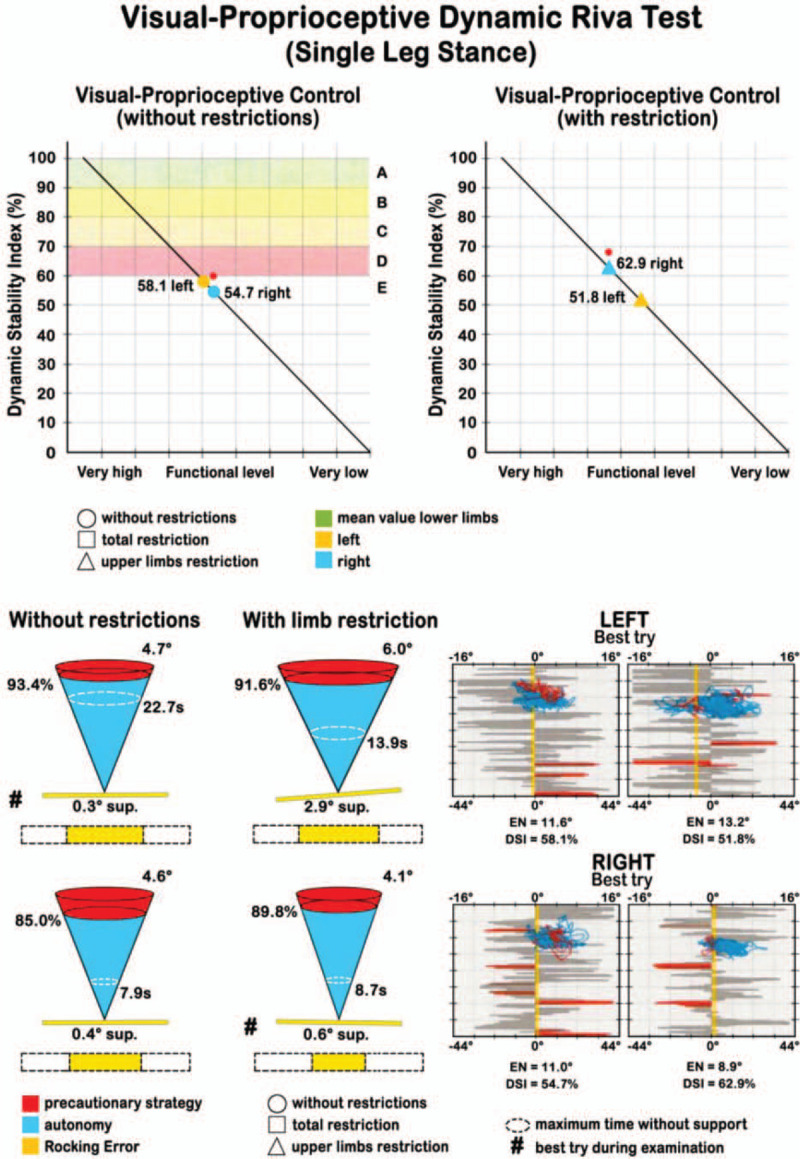
Graphic evaluation of the Dynamic Stability Index (DSI). Values were expressed in % with a color code (red, yellow and green): the lower the DSI, the higher the probability of re-injury in the future. Other information derived by the representation of the DSI as cones, similarly to the static test.

Two graphs evaluating the functional level of the Dynamic Stability Index (DSI) were produced, with and without restriction (Fig. 5). Values were expressed in percentages and were color- coded (red, yellow and green): the lower the DSI, the higher the probability of re-injury in the future. DSI in the test with restriction is often higher than that without restriction because proprioceptive control systems are reactivated, whose main task is to maintain balance with respect to the more frequently used secondary compensation of the upper part of the body.

### 2.6 Comparison and statistical analysis

In order to compare data from patients to a healthy reference sample of same age and sex, data provided by the Delos company were used both for SSI and DSI. This allowed to evaluate the percentile where each patient is located and, if compared with the control population, their risk of re-injury expressed as high, medium, or low (Figs. 4 and 5). Furthermore, data relative to SSI and DSI of the operated limb test were compared with data from the contralateral limb test.

Due to the small number of patients included in the study, a statistical analysis was not possible and only a qualitative analysis was performed for comparison.

## 3 Results

Of the 11 patients, 6 were females and 5 males, mean age 38.4 ± 12 years (range 20–56), 5 patients operated at the right ankle and 6 at the left 1. Mean BMI was 26.8 ± 4.4. Mean follow-up was 13.4 ± 2.1 months (range 12–18).

The mean value of AOFAS clinical score for subjective clinical-functional evaluation was 90.3/100 (range 65–100, SD 13.3).

At the physical examination, the operated ankle presented a recovered lateral mechanical stability in all patients, both in the anterior drawer test and in the inversion tilt test.

**Figure 6:**
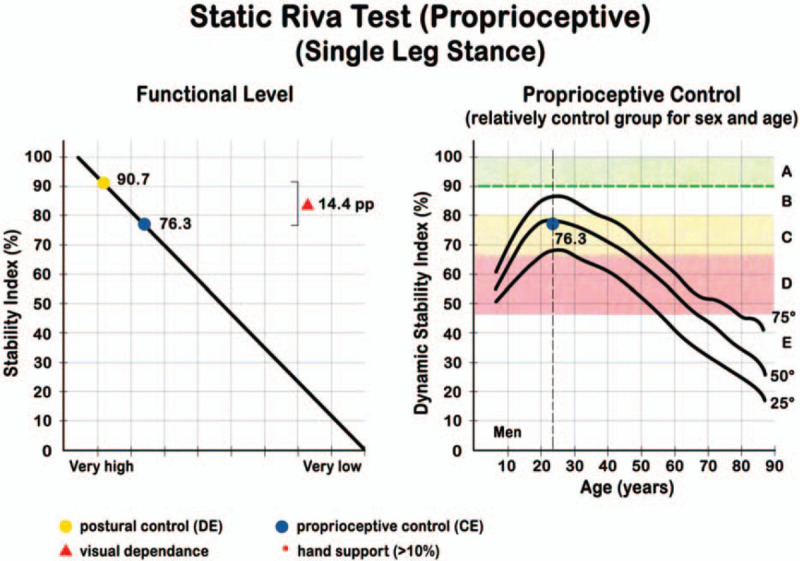
Example of a Static Riva test output for a patient: SSI mean values with closed eyes (*proprioceptive control*) have been positioned in the respective percentile, correlated to a reference healthy sample basing on age and sex. CE = closed eyes, OE = open eyes. Progressive alphabetic letters indicate risk bands. Black curved lines indicate percentiles.

The *static proprioceptive Riva test* evidenced that 7 patients had a *postural control,* with open eyes, higher than 90%, and 4 between 90% and 72.9%. *Proprioceptive control* was expressed by the SSI with closed eyes. The score was expressed in percent, for the operated limb, for the contralateral limb and as mean value between the 2. Four patients had a proprioceptive control below the 25th percentile, 2 patients between 25th and 50th, 2 at 50th, 2 between 50th and 75th, and 1 patient had a value at 75th. None of the subject registered a value above 75th percentile (Fig. 6).

SSI score with open eyes was compared between operated and contra-lateral leg: mean SSI (OE) was 87.7% (±7.6) in the operated leg and 90.4% (±6.1) in the contra-lateral, at the same conditions. SSI mean value (CE) was 64.5% (±11.2) in the operated leg and 61.6% (±16.8) in the contra-lateral.

In the *Dynamic Riva test,* the mean DSI, which represent the visual-proprioceptive control in single leg stance without restrictions was 56.2% (±14.6) in the operated leg and 56.8% (±10.6) in the contra-lateral. DSI with restricted upper limbs, had a mean value of 56.3% (±11.4) in the operated leg and 58.1% (±11.9) in the contra-lateral. Mean values are summarized in Table 1.

**Table 1:**
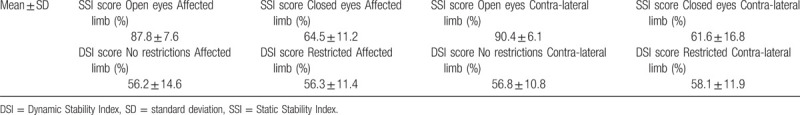
Summary of the mean values registered during the study.

## 4 Discussion

Findings of the present study reveal, at a mean follow up of 13.4 months from capsulo-ligamentous reconstruction in CAI, a clinically stable ankle joint, with an excellent functional scores as measured by the AOFAS and optimal postural control.

The assessment of postural instability and proprioception by means of the DPPS confirms in fact that the Static Stability Index (SSI) when standing on the operated ankle with open eyes is comparable with values when standing on the contralateral limb and within the range of healthy people. This is the most important parameter as it represents the “normality condition”, i.e., what happens in everyday life, where articular stability and the consequent postural stability during the monopodalic stance (for example during walking) are complemented by sight (postural stability).

The Static Stability Index with closed eyes also did not show relevant differences between the operated and the contralateral leg in the test, remaining within the healthy subject reference values.

The Dynamic Stability Index (DSI) provided by the trials on the rocking board also provided comparable values between the operated leg and the contralateral 1, both with and without restriction of the use of upper limbs movement to maintain balance.

Also, the device used in the present study demonstrated to provide reliable parameters, useful to measure postural control and proprioception, as already reported in the literature.^[2,14]^ Although the dynamic balancing assessment methods standing on a force plate are nowadays considered the “gold standard” as measure of postural control,^[16–18]^ the assessment of single leg stance used in the present study for the first time on CAI patients provide a postural and proprioception measure more closed to the condition in which the ankle sprains occur. Unfortunately, no other studies using this device are available for comparison in patients with surgical repair in CAI.

CAI has been related to altered proprioception and poor postural control, both for mechanical and functional instability. Due to this alteration in postural control strategy, in presence of a dysfunctional tibio-tarsal articulation^[1]^ during single leg stance, CAI patients adopt the so-called “hip strategy” to maintain their balance, instead of the correct ankle strategy.^[15]^ Previous studies^[6,12,13]^ though the evaluation of sense of position in CAI patients support the hypothesis that disturbed joint position sense function is a causative factor of functional instability and that proprioceptive malfunction has a role in the development CAI. Re-tensioning the lateral ankle ligaments allows recovering of the proprioceptive function.^[13]^ Considering that surgical reconstruction of ankle ligaments implies a mechanical stabilization of the ankle, results of the present study suggest that as a consequence, postural stability of the operated leg is restored, being comparable to the non-operated limb and to healthy subjects. Hence, surgical repair of capsule and ligaments is effective not only for mechanical ankle instability but also for functional ankle instability, concerning modifications emerging in the neuromuscular system for dynamic support to the ankle.

Certain limitations of the present study need to be discussed. Firstly, due to the small number of patients, a statistical analysis was not carried out, and only a comparative qualitative assessment between the 2 limbs, and with reference data for healthy population was possible. Secondly, no information on the postural and proprioception condition of patients before the surgical intervention was available. Also, no information was available with respect to the sport activity level and the rehabilitation training performed by the patients. Further studies on pre-post surgical intervention postural and proprioception control in CAI patients are guarantees.

In conclusion, with the number of the present study at about 1 year post-operative follow-up, results confirm that surgical repair of ankle external compartment restores proprioceptive and postural control in CAI patients, being comparable with the healthy population of the same age and sex, and with contralateral limb.

## Acknowledgments

Authors would like to thank Dr. Maria Pia Cumani from Anatomical Design School, IRCCS-Rizzoli Orthopaedic Institute (Bologna, Italy) for artwork and figures.

## Author contributions

**Conceptualization:** Silvio Caravelli, Maria Grazia Benedetti.

**Data curation:** Simone Massimi.

**Investigation:** Massimiliano Mosca, Silvio Caravelli, Giuseppe Catanese.

**Methodology:** Massimiliano Mosca.

**Software:** Mario Fuiano.

**Supervision:** Maria Grazia Benedetti.

**Visualization:** Giuseppe Barone, Laura Bragonzoni.

**Writing – original draft:** Silvio Caravelli.

**Writing – review & editing:** Maria Grazia Benedetti.

Silvio Caravelli orcid: 0000-0001-9092-225X.
